# Coronary angiography using spectral detector dual-energy CT: is it the time to assess myocardial first-pass perfusion?

**DOI:** 10.1186/s41747-022-00313-w

**Published:** 2022-12-08

**Authors:** Tommaso D’Angelo, Simon Martin, Antonino Micari, Christian Booz, Alexandra Steyer, Alfredo Blandino, Ludovica R. Lanzafame, Vitali Koch, Giorgio Ascenti, Silvio Mazziotti

**Affiliations:** 1 Department of Biomedical Sciences and Morphological and Functional Imaging, University Hospital Messina, Messina, Italy; 2 Department of Radiology and Nuclear Medicine, Erasmus MC, Rotterdam, The Netherlands; 3 Division of Experimental Imaging, Department of Diagnostic and Interventional Radiology, University Hospital Frankfurt, Frankfurt am Main, Germany

**Keywords:** Computed tomography angiography, Coronary artery disease, Coronary stenosis, Myocardial perfusion imaging, Tomography (x-ray computed)

## Abstract

Coronary computed tomography angiography (CCTA) represents a common approach to the diagnostic workup of patients with suspected coronary artery disease. Technological development has recently allowed the integration of conventional CCTA information with spectral data. Spectral CCTA used in clinical routine may allow for improving CCTA diagnostic performance by measuring myocardial iodine distribution as a marker of first-pass perfusion, thus providing additional functional information about coronary artery disease.

## Keypoints

- The diagnostic performance of coronary computed tomography angiography may be expanded by using spectral data.
- Iodine perfusion maps can functionally assess the impact of coronary stenosis.
- Myocardial iodine distribution may be used as a marker of myocardial perfusion.

## Main text

The use of coronary computed tomography angiography (CCTA) has seen a major increase in the last decade as a first-line noninvasive technique for evaluation of coronary artery disease, in patients with low or moderate risk of disease. Nowadays, CCTA is able to detect coronary stenoses with excellent sensitivity and negative predictive value [1]. Additionally, it allows for plaque characterization and noninvasive assessment of the hemodynamic impact of coronary stenosis by obtaining multivessel calculated fractional flow reserve (cFFR) [2].

Technological advances have furtherly broadened the potential of this technique for cardiovascular applications by the introduction of spectral imaging [3, 4]. In particular, it is possible to derive the material attenuation coefficients by exploiting different x-ray energy levels, in order to obtain information that cannot be evaluated on conventional single-energy scans. Moreover, thanks to the unique absorption characteristics of elements such as iodine, it is possible to generate colour-coded maps that reflect the iodine distribution during the first-pass perfusion of organs and tissues. Several studies have shown that dual-energy imaging may allow the use of iodine distribution maps as a surrogate marker of myocardial perfusion both in phantom and human studies, potentially providing additional functional information for the assessment of coronary artery disease [5–7].

The main approaches used to obtain dual-energy imaging are dual-source platforms, made by two x-ray tubes and detectors that simultaneously acquire data; voltage-switching platforms, where a single x-ray tube rapidly switches between low- and high-energy levels; and dual-layer spectral detector platforms, which allow to collect low-energy and high-energy data at the same time with a single detector, composed by two scintillation layers. The sensitivity of each layer is determined by its composition: low-energy x-rays are detected by an yttrium-based top layer, whereas a gadolinium-oxysulfide bottom layer is sensitive to high-energy x-rays, providing a spectral dataset [8].

At our centre, we have been performing CCTA on a dual-layer DECT system (IQon, Philips Healthcare, Best, The Netherlands) for almost a year. Based on our experience, dual-layer dual-energy computed tomography (DECT) has proved to be a robust technique to assess first-pass myocardial iodine distribution during CCTA, by measurement of iodine maps on a per-segment basis [9]. In fact, myocardial segments with reduced first-pass iodine distribution might be related to the reduction of normal myocardial perfusion due to coronary artery stenosis. The recent advent of computed fractional flow reserve (cFFR) in CCTA studies has been shown to relate well with invasive fractional flow reserve (FFR) for the assessment of hemodynamically significant coronary artery stenoses, also allowing for a multivessel analysis [2, 10, 11].

We measured noninvasive cFFR based on our spectral CCTA datasets with dedicated software (CT cFFR version 3.5, Siemens Healthineers, Erlangen, Germany). Before the execution of CCTA, patients were medicated with a sublingual vasodilator agent (isosorbide dinitrate, sublingual tablets, 5 mg) and patients whose heart rate was greater than or equal to 70 beats per minute were administered β-receptor blocker (Esmolol hydrochloride injection, Baxter, Deerfield, Illinois). The iodine concentration of all myocardial segments was measured by vendor-recommended software (IntelliSpace Portal version 8.0, Philips Healthcare), and the segment with the least value was reported and assigned to left anterior descending artery (LAD), left circumflex (LCx), and right coronary artery (RCA), according to the standardised myocardial segmentation [9].

Initial results on a small cohort of patients who underwent spectral CCTA and presented a pathological cFFR (*i.e.,* ≤ 0.75) show a difference in iodine distribution between normally perfused and ischemic myocardial segments (Table 1) [11]. Our preliminary results also show that iodine distribution in myocardial segments irrorated by coronary arteries affected by non-hemodynamically significant stenoses did not differ significantly from the global myocardial iodine distribution (Table 1).

**Table 1:** Per-vessel analysis of maximal coronary stenosis, cFFR, and first-pass myocardial iodine distribution based on spectral CCTA

	Sex	Age (years)	Vessel	Maximal stenosis	cFFR	Minimal segmental iodine concentration (mg/mL)	Mean global iodine concentration (mg/mL)	Segmental *versus* global iodine concentration (*p* values) *
Patient 1	Male	57	LAD	54%	0.68	0.92	1.68	**0.001**
			LCx	51%	0.96	1.61		0.516
			RCA	10%	0.87	1.52		0.136
Patient 2	Female	63	LAD	37%	0.74	1.18	1.66	**0.001**
			LCx	21%	0.95	1.51		0.118
			RCA	40%	0.88	1.65		0.972
Patient 3	Male	74	LAD	75%	0.56	1.58	2.03	**0.001**
			LCx	45%	0.96	1.93		0.134
			RCA	48%	0.88	2.02		0.837
Patient 4	Male	75	LAD	70%	0.67	1.61	2.06	**0.001**
			LCx	58%	0.87	1.99		0.136
			RCA	68%	0.89	1.97		0.059

*Statistical analysis was performed using Graphpad Prism version 9.1 (GraphPad Software, San Diego, CA, USA). The differences between the minimal segmental iodine concentration and the mean global iodine concentration were performed by one-sample *t* test. Significant values are in bold. *LAD* Left anterior descending artery, *LCx* Left circumflex artery*, RCA* Right coronary artery*, cFFR* Calculated fractional flow reserve

**Fig. 1:**
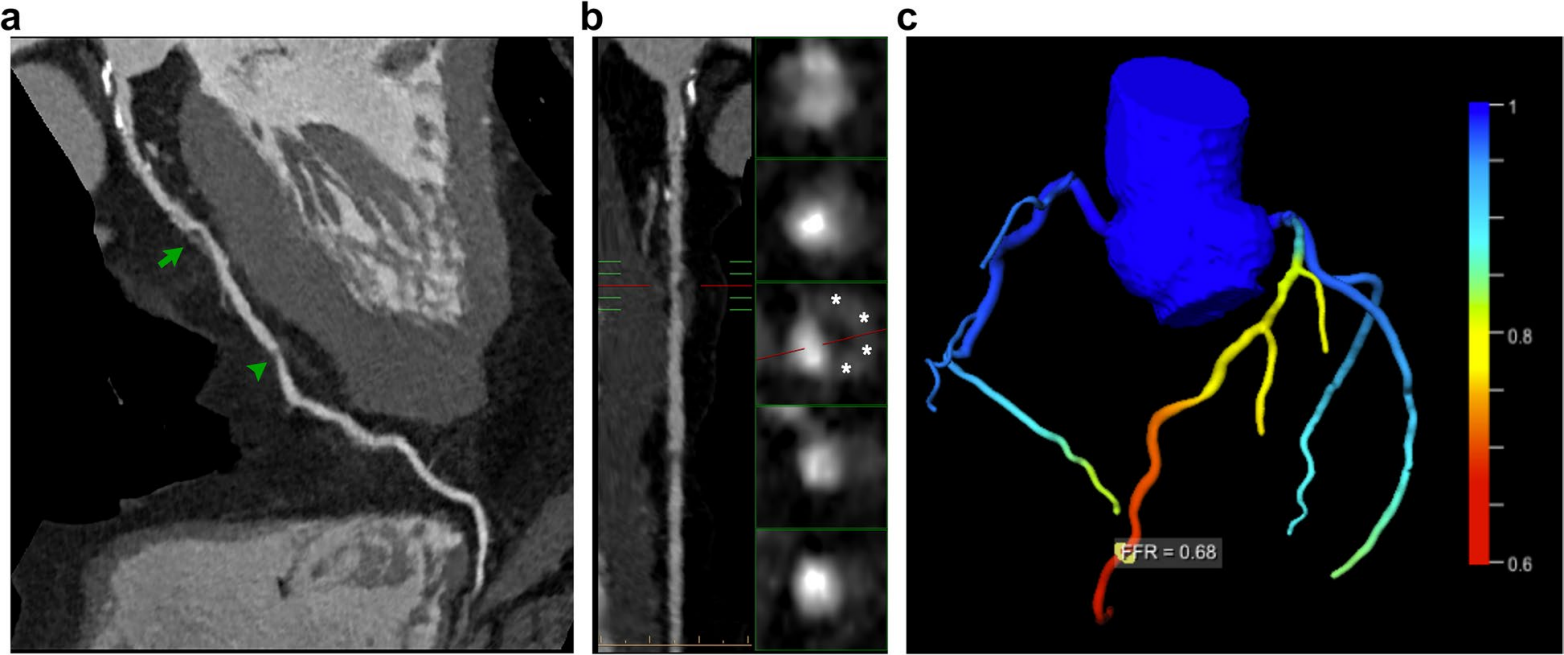
CCTA MPR images of the LAD artery (**a**, **b**) and volume rendering reconstructions of coronary tree with cFFR assessment (**c**) performed on patient 1 (see Table 1). Conventional CCTA images show different mixed plaques along the left main artery and LAD artery, with noncalcified plaques in the LAD proximal segment (*arrow*), which causes a 54% stenosis, and mid-segment (*arrowhead*), which causes a 31% stenosis (**a**). The orthogonal MPR sections show the napkin-ring sign (*asterisks*), which is a marker of plaque vulnerability (**b**). Multivessel cFFR measurement shows a hemodynamically significant stenosis of LAD (**c**). *CCTA* Conventional coronary computed angiography, *cFFR* Calculated fractional flow reserve, *LAD* Left anterior descending, *MPR* Multiplanar reformatted

**Fig. 2:**
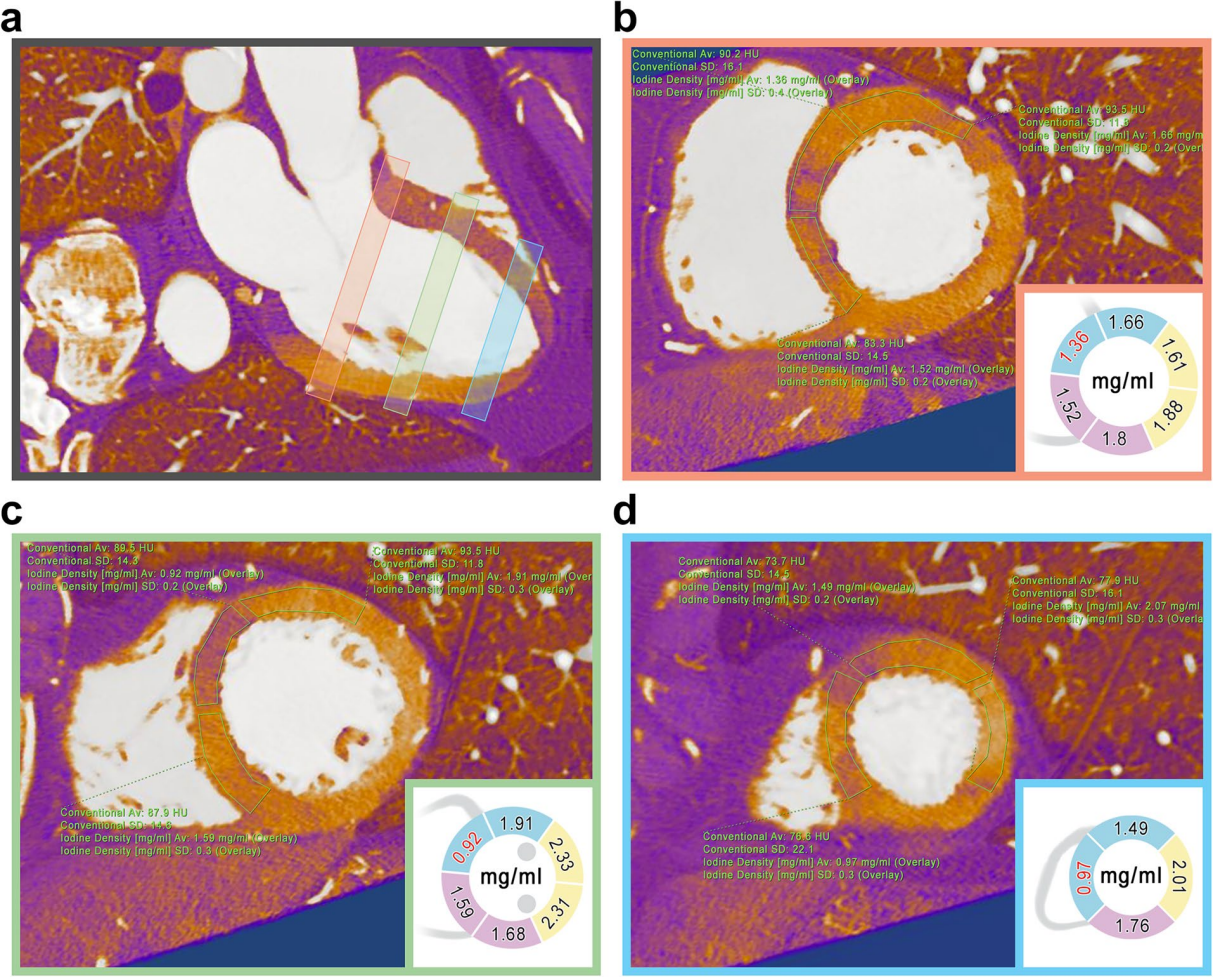
Spectral CCTA MPR images of the heart with iodine density map overlay performed on patient 1 (see Table 1). Myocardial iodine concentration was assessed by means of polygonal ROIs placed in each short-axis segment (**b**, **c**, **d**) accordingly to the Standardized Myocardial Segmentation and Nomenclature of the American Heart Association. Iodine concentration was reduced in the anteroseptal myocardial segments, as visible on the three-chamber long-axis view (**a**). ROIs placed on each myocardial segment, either on basal (**b**), mid (**c**), and apical (**d**) short-axis slices, confirm reduced iodine distribution of the myocardial segments perfused by LAD artery (blue), compared to RCA (purple) and LCx artery (yellow). *CCTA* Conventional coronary computed angiography, *LAD* Left anterior descending, *LCx* Left circumflex, *MPR* Multiplanar reformatted, *RCA* Right coronary artery, *ROI* Region of interest

**Fig. 3:**
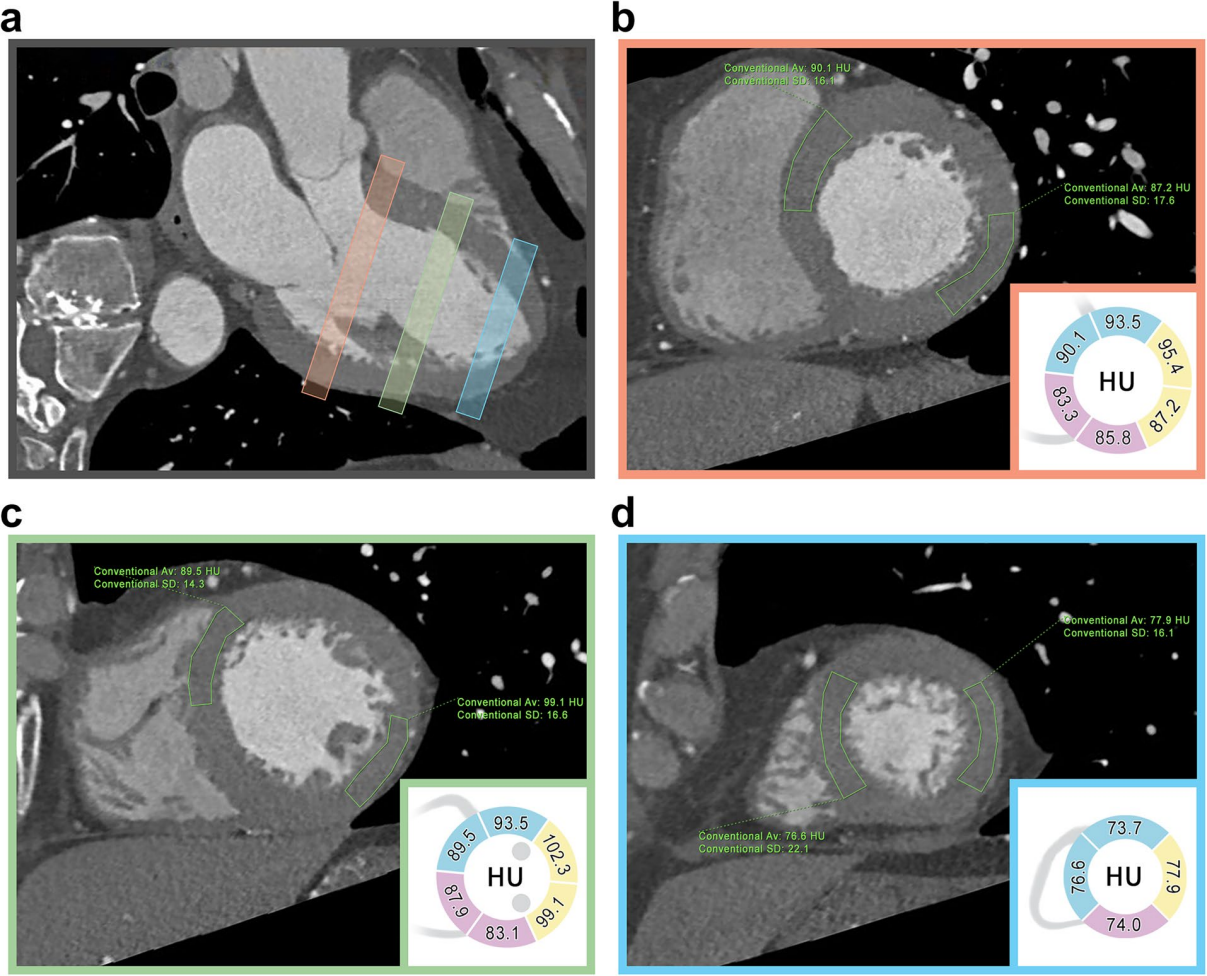
Conventional CCTA MPR images of the heart performed on patient 1 (see Table 1). Myocardial attenuation was assessed by means of polygonal ROIs placed in each short-axis segment (**b**, **c**, **d**) accordingly to the Standardized Myocardial Segmentation and Nomenclature of the American Heart Association. Myocardial attenuation does not show any difference between anteroseptal and inferolateral segments, as visible on the three-chamber long-axis view (**a**). ROIs placed on each myocardial segment, either on basal (**b**), mid (**c**), and apical (**d**) short-axis slices, show similar HU values among myocardial segments perfused by LAD arstery (blue), RCA (purple), and LCx artery (yellow). *CCTA* Conventional coronary computed angiography, *LAD* Left anterior descending, *LCx* Left circumflex, *MPR* Multiplanar reformatted, *RCA* Right coronary artery, *ROI* Region of interes

As a sample case, we show patient 1 (see Table 1), who underwent spectral CCTA that showed the presence of moderate stenosis (54%) in the proximal tract of the LAD, with signs of plaque vulnerability such as “napkin-ring sign” and positive remodeling (Fig. 1a, b). CCTA also showed a mild stenosis (31%) at the mid tract of LAD and a moderate stenosis of LCx, caused by a noncalcified plaque. The cFFR performed on the same dataset showed a pathological reduction for LAD (0.68), confirmed by invasive FFR (0.73) executed prior to revascularization, while the FFR values for the other vessels were normal (Fig. 1c). Similarly, iodine density maps evaluated on a per-vessel basis revealed a significant reduction of iodine distribution (mg/mL) within the myocardial segments perfused by LAD (Fig. 2), compared to global iodine myocardial distribution [9]. In particular, the iodine concentration at the basal anteroseptal segment, the least perfused, was lower compared to the average iodine concentration in all myocardial segments, with values of 0.92 mg/mL *versus* 1.68 mg/mL, respectively (*p* < 0.001). On the other hand, no difference in terms of myocardial attenuation (HU) was observed (Fig. 3). Conventional images did not show any segmental differences in terms of myocardial attenuation, with values of 85.4 HU *versus* 87.4 HU, respectively (*p* = 0.731).

Our initial experience indicates that iodine distribution maps may provide an additional functional parameter to assess coronary artery disease, which may further increase CCTA diagnostic performance.

To the best of our knowledge, there are limited studies in the scientific literature that have evaluated the feasibility of myocardial perfusion imaging by means of dual-energy CT iodine distribution, and only a few have assessed the potential of spectral-detector platforms, using FFR as the reference standard [12–14].

This initial data does not currently allow for defining a cutoff value to distinguish between ischemic *versus* nonischemic segments, due to the limited sample size. Additional prospective multicentre studies with larger patient cohorts are needed to establish whether iodine distribution maps obtained from spectral datasets can be used as a surrogate marker of myocardial first-pass perfusion.

We hypothesise that iodine perfusion maps can help distinguish between hemodynamically significant and nonsignificant coronary stenoses, improving the diagnostic performance of CCTA.

## Data Availability

The anonymised dataset supporting the conclusions of this article is available upon request.

## Abbreviations

CCTA: Coronary computed tomography angiography
cFFR: Calculated FFR
DECT: Dual-energy computed tomography
FFR: Fractional flow reserve
LAD: Left anterior descending
LCx: Left circumflex
RCA: Right coronary artery
